# Impaired object-location learning and recognition memory but enhanced sustained attention in M2 muscarinic receptor-deficient mice

**DOI:** 10.1007/s00213-018-5065-7

**Published:** 2018-10-16

**Authors:** Carola Romberg, Susan Bartko, Jürgen Wess, Lisa M. Saksida, Timothy J. Bussey

**Affiliations:** 1 Department of Psychology, University of Cambridge, Cambridge, CB2 3EB UK; 2 Wellcome Trust and MRC Behavioural and Clinical Neuroscience Institute, University of Cambridge, Cambridge, CB2 3EB UK; 3 Department of Psychology, Research Unit Biological Psychology, Ludwig-Maximilians-University, Munich, Germany; 4 Molecular Signaling Section, Laboratory of Bioorganic Chemistry, National Institute of Diabetes and Digestive and Kidney Diseases, National Institutes of Health, Bethesda, MD 20892 USA; 5 Brain and Mind Institute, Western University, London, ON Canada; 6 Molecular Medicine Research Laboratories, Robarts Research Institute & Department of Physiology and Pharmacology, Schulich School of Medicine & Dentistry, Western University, London, ON Canada

**Keywords:** M2, Muscarinic, Acetylcholine, Learning, Memory, Attention, Recognition, Paired-associates learning

## Abstract

**Rationale:**

Muscarinic acetylcholine receptors are known to play key roles in mediating cognitive processes, and impaired muscarinic cholinergic neurotransmission is associated with normal ageing processes and Alzheimer’s disease. However, the specific contributions of the individual muscarinic receptor subtypes (M1–M5) to cognition are presently not well understood.

**Objectives:**

The aim of this study was to investigate the contribution of M2-type muscarinic receptor signalling to sustained attention, executive control and learning and memory.

**Methods:**

M2 receptor-deficient (M2^−/−^) mice were tested on a touchscreen-operated task battery testing visual discrimination, behavioural flexibility, object-location associative learning, attention and response control. Spontaneous recognition memory for real-world objects was also assessed.

**Results:**

We found that M2^−/−^ mice showed an enhancement of attentional performance, but significant deficits on some tests of learning and memory. Executive control and visual discrimination were unaffected by M2-depletion.

**Conclusions:**

These findings suggest that M2 activation has heterogeneous effects across cognitive domains, and provide insights into how acetylcholine may support multiple specific cognitive processes through functionally distinct cholinergic receptor subtypes. They also suggest that therapeutics involving M2 receptor-active compounds should be assessed across a broad range of cognitive domains, as they may enhance some cognitive functions, but impair others.

## Introduction

Cholinergic signalling in the brain exerts control over fundamental cognitive processes such as memory, attention and executive control (Hasselmo and Sarter 2011; Ballinger et al. 2016). Moreover, cognitive deficits occurring in the course of normal ageing, and particular in Alzheimer’s disease, can be attributed at least in part to deficiencies of the cholinergic system (Drachman and Leavitt 1974; Bartus et al. 1982; Lawrence and Sahakian 1995), which is therefore a prime target for pharmacological intervention. Yet, it is still unclear how coordinated acetylcholine (ACh) release from basal forebrain (and other) projection neurons to cortical and subcortical structures exerts control over cognitive processes. Previous studies and models suggest that the cognitive effects of ACh may depend on the timing of ACh release, the dose and the brain region. Accordingly, tonic and phasic cholinergic components may dynamically regulate the state, and thus function, of local networks (Hasselmo and Sarter 2011). For example, strong cholinergic stimulation during active wake and the resulting cortical/hippocampal circuit dynamics may make afferent sensory input selectively available for respective further processing, such as working memory, top-down control or long-term memory encoding. In contrast, low cholinergic tone and/or the activation of different cholinergic inputs/receptors during quiet wake or sleep may activate recurrent network dynamics necessary for internal processing such as memory consolidation (Hasselmo and McGaughy 2004). Thus, at a given time/state, ACh release may have variable effects across cognitive domains.

Furthermore, unequivocal conclusions about cholinergic function are hindered by the diverse range of functionally distinct receptor subtypes, broadly divided into nicotinic (nAChR) and muscarinic (mAChR) subtypes, which are both widely distributed across the brain. Moreover, mAChRs are metabotropic receptors found in at least five subtypes, M1–M5.

In the present study, we focus on the M2 mAChR subtype and its role for cognition. M2 receptors are thought to function mostly as pre-synaptic autoreceptors, acting as a brake on neurotransmitter release (Thiele 2013). Thus, M2 receptors are a promising target for pharmacological intervention, since M2 receptor blockade may boost neurotransmission compromised in neurodegenerative disease, such as AD and Parkinson’s disease (Clader and Wang 2005; Koch et al. 2005; Langmead et al. 2008). However, the contribution of M2 receptors to healthy cognitive control is still unclear, because previous studies were restricted by the lack of subtype-specific receptor ligands and failed to test multiple cognitive domains such as memory, executive control and attention. Yet, the latter factor may be particularly important; for example, apparent memory deficits—even delay-dependent deficits—can be due to a failure to attend to and therefore adequately encode the to-be-remembered items, rather than a memory impairment per se (Sarter et al. 2003; Romberg et al. 2012).

Thus, we sought to minimise the problems of receptor and cognitive specificity by testing mutant mice with a targeted deletion of the M2 receptor gene (M2^−/−^) (Gomeza et al. 1999; Bainbridge et al. 2008) on a battery of touchscreen tests. Multiple aspects of memory, requiring distinct neural structures, were tested with three memory tasks (Horner et al. 2013): visual discrimination and reversal learning, paired associates learning (PAL) and spontaneous object recognition with real-world objects (SOR, Winters et al. 2004; Forwood et al. 2005). Sustained attention and executive control were assessed with the 5-choice serial reaction time task (5-CSRTT, Mar et al. 2013).

## Materials and methods

### Animals and housing

Mice with a targeted deletion of the M2 muscarinic receptor gene on a mixed 129/SvEv x CF1 background (Gomeza et al. 1999) had previously been backcrossed onto the C57BL/6 strain (Taconic, Germantown, New York) for 10 successive generations (Bainbridge et al. 2008). Heterozygous M2^+/−^ founder mice were shipped from the National Institute of Mental Health to Cambridge, UK. M2^−/−^, M2^+/−^ and wild-type (M2^+/+^) mice were generated from M2^+/−^ x M2^+/−^ matings on site. Testing cohorts consisted of male M2^−/−^ and wild-type M2^+/+^ littermates.

Mice to be used for behavioural studies were housed in Cambridge in small groups of 4–7 mice per cage under standard conditions, kept in a temperature-controlled room (22 °C) with diurnal light conditions (12 h light; 12 h dark), with food and water ad libitum. Behavioural testing started when mice were 9 months old. Throughout testing, mice were maintained at or above 85% of their free-feeding weight using a restricted feeding regime. Mice were tested by experimenters blind to genotype, 5 days a week during the light phase. Cohort 1 originally contained 8 M2^−/−^ and 10 wild-type male mice and was tested on “Visual Discrimination and Reversal” and paired associates learning (PAL). Cohort 2 contained 8 M2^−/−^ and 6 wild-type mice and was tested on the 5-choice serial reaction time task (5CSRTT). Cohort 3 contained 6 wild-type and 6 M2^−/−^ mice and was tested on the object recognition paradigm (OR). All experiments were conducted in agreement with Home Office regulations under the Animal (Scientific Procedures) Act 1986.

### Testing chambers

The touchscreen operant chambers used for this study were the same as previously described (Romberg et al. 2011). They consisted of a rectangular 21.6 cm × 17.8 cm × 12.7 cm testing chamber with clear Perspex side walls, a touch-sensitive screen at the front and an illuminated pellet receptacle, fitted with head entry detectors, located centrally on the rear wall (Med Associates Inc., Vermont, USA). The receptacle was attached to a dispenser filled with 14 mg sucrose pellets. The chamber also contained a 3 W house light, a tone generator and a fan (for ventilation and to mask external noise). The test chamber and screen were placed in a wooden sound-attenuating box. For each task, the touchscreen was covered by a task-specific black Perspex mask with windows, through which the stimuli could be displayed and the mice could make nose pokes (Horner et al. 2013; Mar et al. 2013; Oomen et al. 2013). The mask prevented accidental triggering of the screen by the mouse. Stimulus presentation and recording of results were controlled by custom written software (mouseCAT, L.M. Saskida and C. Romberg).

### Shaping for touchscreen tasks

Before starting the cognitive tasks, mice received a series of pre-training stages in order to learn to touch a stimulus displayed on the screen for a food reward, as previously published (Bartko et al. 2011b; Romberg et al. 2011; Mar et al. 2013). Initially, mice were habituated to the chambers for 1–3 days and allowed to freely consume sucrose pellets delivered in the reward receptacle. In the following phase, mice learned to associate pellet delivery with stimulus disappearance, tone generation and illumination of the magazine light. Mice had to eat the pellet to initiate the next trial (criterion, 30 pellets consumed within 30 min). Subsequently, mice were trained to nose-poke a stimulus (displayed in either window) for reward (criterion, 30 trials within 30 min, 5 s inter-trial interval). After nose-poking had been learned, the mouse was required to initiate presentation of the stimulus by head entry into the pellet receptacle, indicated by a light in the receptacle (criterion, 30 trials within 30 min). In a final shaping procedure, nose-pokes to the blank window were followed by a 5 s house-lights off period to mark an incorrect response. Following this dark period, a correction trial took place whereby the stimulus was presented again on the same side until a correct response was made. Trials were repeated until mice reached criteria of 90% correct (not including correction trials).

### Experiment 1: visual discrimination and reversal

**Fig. 1:**
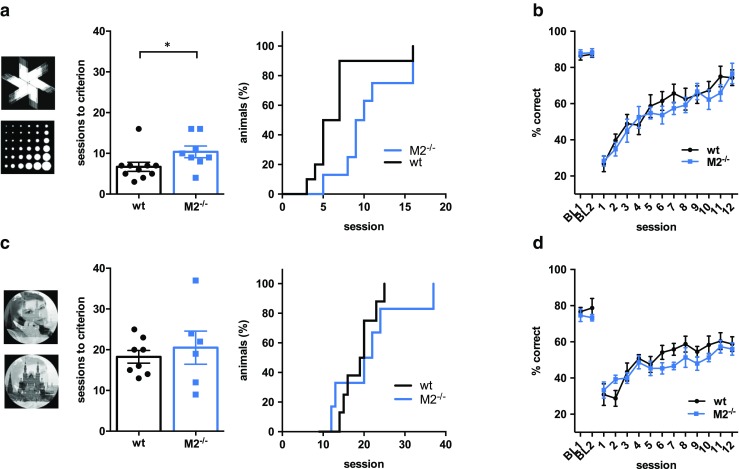
Lack of M2 receptors has different effects on visual discrimination and reversal learning. **a** Acquisition of a visual discrimination task with computer graphic stimuli. Left panel: fan and marble stimuli used for the initial discrimination task. Middle panel: M2^−/−^ mice (*n* = 8) required more sessions to reach criterion (> 80% correct) than wild-type mice (*n* = 10). Right panel: cumulative percentage of animals that had reached criterion across sessions (same data as in middle panel). **b** Baseline performance levels (BL) and accuracies after reversal of task contingencies for the two graphic stimuli (sessions 1–12) were similar in both genotypes (wild type, *n* = 10; M2^−/−^, *n* = 8). **c** Acquisition of a perceptually more demanding visual discrimination paradigm with morphed photographic stimuli (left). The mean number of sessions required to reach criterion (middle) and the cumulative percentage of animals that had reached criterion (right) were unaffected by M2 depletion (wild type, *n* = 8; M2^−/−^, *n* = 6). **d** Baseline performance and accuracies after reversal of task contingencies for the two photographic stimuli were similar in both genotypes (wild type, *n* = 8; M2^−/−^, *n* = 6). Data are represented as mean ± sem

Cohort 1 was tested on two versions of the visual discrimination and reversal task, a test of visual discrimination (acquisition), followed by a test of behavioural flexibility (reversal). Task procedures were followed as previously described (Romberg et al. 2011; Mar et al. 2013). The touchscreen was covered with a black Perspex mask, with two display/response windows. Mice initiated each trial with a nose poke into the pellet receptacle. This caused the simultaneous presentation of two distinct picture stimuli (one in each window). In the first version of the task (experiment 1 a), perceptual demand was relatively low, and the black and white stimuli were relatively easy to discriminate (fan and marble, Fig. 1a). In the second version of the task (experiment 1 b), perceptual demand was higher, and the stimuli comprised morphed photographic images with feature overlap (Fig. 1c). In both versions of the task, one stimulus (S+) was designated ‘correct’ and the other (S−) ‘incorrect’. The side on which the S+ was displayed was determined pseudorandomly for each trial.

In both task versions, nose pokes to the S+ were rewarded with a tone, lighting of pellet receptacle and a sucrose pellet. Pellet collection initiated the start of a 30-s inter-trial interval in which the magazine light was off and initiation of the next trial was not possible. After 30 s, the pellet receptacle lit up and the subsequent trial could be initiated.

Any pokes to the S− resulted in a 5-s house-light off period, followed by a 30-s inter-trial interval and then a ‘correction trial’. In a correction trial, when the mouse initiated the trial, the two pictures were presented in the same left-right configuration as on the previous, incorrect trial. Correction trials were repeated until a correct response was made. The session finished after completion of 30 trials (excluding correction trials) or 1 h. On reaching a criterion of at least 80% correct over two consecutive sessions, mice stopped testing, to avoid overtraining, until all mice had either reached criteria or completed 40 sessions. Group means of the number of sessions required to reach criterion of wild-type and M2^−/−^ mice were subjected to an independent-samples *t* test.

After all mice had reached criterion or completed 40 sessions, those that had reached criterion were given 4 more sessions, over 4 consecutive days, to ameliorate any differences in performance level caused by the mice reaching criterion at different times. Accuracy over the 4 sessions just before reversal was taken as baseline. Baseline response latencies (delay between trial initiation and screen response) were also analysed. For reversal learning, exactly the same procedure was carried out as for the initial visual discrimination except the contingencies were switched so that S− became the correct rewarded response and S+ the incorrect punished response.

Group means of choice accuracy (%) were subjected to a repeated measures ANOVA with genotype as between-subjects factor and session as within-subject factor. All statistical analyses were conducted with a significance level of *p* < 0.05.

### Experiment 2: paired-associate learning

**Fig. 2:**
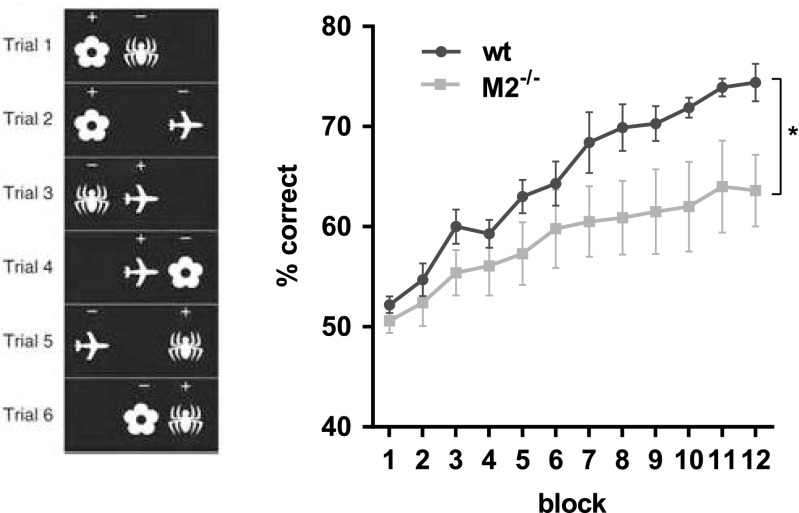
M2 receptor deficiency impairs object-place location learning in the PAL task. Mice had to learn the correct location (left, middle, right) for each of the three objects (flower, spider, plane). The 6 possible trial types are shown on in the panel on the left. The correct stimulus (flower-left, plane-middle, spider-right) is marked with a ‘+’. M2^−/−^ mice (*n* = 8) acquired the PAL task significantly more slowly than wild-type mice (*n* = 9) and never reached the same performance levels (right panel). Each block consisted of 5 sessions. Data are presented as mean ± sem. * Main effect of genotype, *p* < 0.05

Five days after finishing the visual discrimination and reversal task, cohort 1 mice (one animal had to be excluded to an eye infection) were tested on the automated touchscreen PAL task, a test of visuospatial memory that has previously revealed deficits after pharmacological manipulation of muscarinic receptors (Bartko et al. 2011b). The task was performed as previously described (Bartko et al. 2011b). At the beginning of a session, the mouse was required to initiate the first trial. Then, a pair of 2 out of 3 stimuli (flower, airplane and spider; Fig. 2a) would appear on the screen, in two of the three locations (left, middle and right), but only one stimulus would appear in its correct location (S+), whereas the other would appear in an incorrect location (S−), i.e., the flower was rewarded only when presented in the left location, the airplane was rewarded only when presented in the middle location, and the spider was rewarded only when presented in the right location. Thus, there were six possible trial types (Fig. 2a). A nose-poke to the correct S+ resulted in a tone, magazine light and reward pellet. Incorrect responses resulted in a 5-s time-out period, followed by correction procedure. Nose-pokes to response windows in which no stimulus was presented had no programmed consequences. The inter-trial interval (ITI) during the task was fixed at 20 s. In a testing session, mice were given 1 h to complete 36 trials (each trial type occurred six times, and the same trial type was never presented more than twice in a row).

Group means of accuracy (percent correct) were submitted to a RM ANOVA with group as between-subjects factor and session as within-subject factor. Furthermore, mean response latencies (delay between trial initiation and screen touch) of the last two blocks were compared by a one-way ANOVA with genotype as between-subjects factor. All statistical analyses were conducted with a significance level of *p* < 0.05.

### Experiment 3: 5-choice serial reaction time task

Cohort 2 was tested on the 5CSRTT, a continuous performance test measuring elements of sustained attention and response control (Robbins 2002; Mar et al. 2013). Pre-training and task procedure were carried out as previously described (Romberg et al. 2011; Bartko et al. 2011a). Mice were trained to respond to brief flashes of light pseudorandomly displayed in one of the five spatial locations on the touchscreen. Mice were tested 5–6 days a week, 50 trials a day (or up to 1 h). Each trial commenced with the illumination of the magazine light. However, a nose poke to the magazine did not result in the immediate display of a stimulus. Instead, the stimulus was delivered after a variable 5–10 s delay (the delay period), during which the animal was required to attend to the screen. If an animal prematurely touched the screen during this delay, the response was recorded as premature and followed by a 10-s time-out period (house light off, magazine inactive). The time-out was followed by a 20-s ITI (house light on, magazine inactive), after which the illumination of the magazine light signalled the onset of the next trial. The stimulus duration was initially set to 4 s, followed by a ‘limited hold’ period of 5 s, during which the stimulus was absent but the animal was still able to respond to the location. Responses during stimulus presence or the limited hold period were recorded either as correct (response to the stimulus window) or incorrect (response to any other window). A correct choice was rewarded with a tone and pellet delivery, indicated by the illumination of the magazine light. Reward collection turned the magazine light off and triggered an ITI of 20 s. An incorrect response was followed by a 10-s time-out (house light off), extended by a 20-s ITI (house light on). A failure to respond to any window during the combined stimulus display and limited hold period was recorded as an omission and punished with a 10-s time-out, followed by a 20-s ITI. In addition, perseverative responses to the screen after premature (during time-out), correct (before collecting the reward) and incorrect (during time-out) choices were also recorded.

Once the performance of a mouse stabilised at 4 s stimulus duration (> 80% accuracy, < 20% omissions for 3 consecutive days), the stimulus duration was reduced to 2 s. After reaching criterion with the 2-s stimulus, animals were tested for two more days. The mean measures of those 2 days were used to assess baseline performance. Group means of baseline response accuracy (percent correct), omissions (percent), premature responses (percent) and perseverative responses (per choice), response latencies and reward collection latencies were submitted to a one-way ANOVA with genotype as between-subjects factor.

After stable-baseline performance, animals were challenged with an increased attentional demand (probe trials) by reducing the stimulus duration to 1 s, 0.8 s and 0.6 s. To control for order effects, the sequence of stimulus durations presented to each animal in a group was randomised in a Latin-square design. Each animal performed 2 consecutive days at a given stimulus duration and was then moved back onto a 2-s stimulus duration for 2 days, or until it re-attained criterion (> 80% accuracy, < 20% omissions).

Group means of response accuracy (percent correct), omissions (percent), premature responses (%) and perseverative responses (per choice), response latencies and reward collection latencies were submitted to a repeated measures ANOVA with genotype as between-subjects factor and stimulus duration as within-subject factor. All statistical analyses were conducted with a significance level of *p* < 0.05.

### Experiment 4: spontaneous object recognition

Cohort 3 was tested on a spontaneous object recognition task in a Y-maze (Winters et al. 2004; Forwood et al. 2005; Bartko et al. 2007). The task is motivated by animals’ inherent tendency to explore novel objects, and measures recognition memory by the degree of exploration of novel compared to familiar objects. Each test session started with a sample phase, followed by a 1-min or 3-h delay and ended with a choice phase. Before each sample phase, the mouse was placed in the start arm of the Y-maze. By opening a sliding door that separated the start arm from the 2 choice arms, the sample phase was started and the animal was left to explore two identical sample objects placed at the ends of the 2 other arms, for 5 min. During this period, the experimenter recorded the time an animal explored each of the two objects. Periods when the animal sat on or chewed an object were not counted. After the sample phase, mice were either returned to their home cage for 3 h before the choice phase, or the choice phase started immediately (after a 1-min delay). The choice phase was identical to the sample phase, except that the object pair consisted of the familiar, previously encountered object and one novel object. The side of the novel object was counterbalanced across animals of each group. Each animal received four test sessions per delay condition, separated by at least 24 h, counterbalanced for object pairs and object order across animals. For each session, a preference score (d2) was calculated by dividing the difference between exploration time of the novel object and exploration time of the familiar object by the sum of exploration times of the novel object and the familiar object together. The mean d2 score of all four sessions for each animal and delay was subjected to a RM ANOVA with delay as within-subject factor and genotype as between-subjects factor, followed by post hoc tests for individual group differences, with Bonferroni correction for multiple comparison. All statistical analyses were conducted with a significance level of *p* < 0.05.

## Results

### Experiment 1a: visual discrimination and reversal with simple stimuli

After touchscreen pre-training, wild-type (*n* = 10) and M2^−/−^ (*n* = 8) mice were tested on a visual discrimination and reversal paradigm with black and white stimuli that were perceptually easy to discriminate.

Both groups of mice eventually learned to discriminate between S+ and S−, but M2^−/−^ mice required significantly more sessions to reach criterion (Fig. 1a, independent-sample *t* test, t(17) = 5.17, *p* = 0.023). Plotting the cumulative percentage of animals that had reached criterion across sessions also indicated that the group of M2^−/−^ mice took longer than the wild-type group to reach criterion. However, a log-rank (Mantel-Cox) test returned no significant difference between the fitted curves of the two genotypes (*χ*^2^_3_ = 3.37, *p* = 0.33). Moreover, baseline response accuracies of the last 2 days of acquisition were similar in both groups (Fig. 1b, RM ANOVA with genotype as between-subjects factor and session as within-subject factor, no main effect of genotype (F(1,16) = 0.12, *p* = 0.73) or genotype x session interaction (F(1,16) = 0.01, *p* = 0.97)), confirming that both groups performed equally well before reversal and had formed comparable stimulus-reward associations.

After reversing the task contingencies, response accuracies of wild-type and M2^−/−^ mice dropped to 30.8% and 30.2%, respectively. They subsequently increased in a similar manner, indicating no reversal (< 50% accuracy) or re-learning (> 50% accuracy) deficits in M2^−/−^ mice (Fig. 1b, RM ANOVA, no main effect of genotype, F(1,16) = 1.25; *p* = 0.279; main effect of session, F(11,176) = 36.46, *p* < 0.001; no genotype x session interaction, F(11,176) = 1.1, *p* = 0.364).

### Experiment 1b: visual discrimination and reversal with morphed photographic stimuli

In order to increase the perceptual challenge of the visual discrimination, animals were subsequently tested on a new set of photographic stimuli with considerable feature overlap. All but 4 mice (2 wild-type and 2 M2^−/−^ mice, subsequently excluded from analysis and reversal phase) acquired the discrimination task with morphed photographic stimuli within 40 sessions, and both genotypes required a similar number of sessions to reach criterion (Fig. 1c; M2^−/−^, 22.1 sessions ± 4.83; wild-type, 18.3 sessions ± 2.97; t(14) = 0.016, *p* = 0.49). The cumulative frequency of animals reaching criterion across sessions was also similar in both genotypes (Fig. 1c, left panel; Log-rank Mantel-Cox test, *χ*^2^_3_ = 0.46, *p* = 0.92).

After reaching criterion, baseline performance was recorded for 4 more days. A one-way ANOVAs of baseline response latencies (wt, 3128 ± 270 ms; M2^−/−^, 3101 ± 619 ms) and reward collection latencies (wt, 1631 ± 137 ms; M2^−/−^, 1709 ± 90 ms) returned no significant differences (response latency, F(1,12) = 0.002, *p* = 0.97; reward collection latency, F(1,12) = 0.207, *p* = 0.658) suggesting that both groups of mice did not differ in terms of task-relevant motor abilities or motivation. Furthermore, a RM ANOVA of choice accuracies of the last 2 days of baseline performance showed no significant effect of genotype (F(1,12) = 0.81, *p* = 0.39), suggesting that both groups of mice performed equally well before reversal.

Reversing the reward contingencies of the stimuli caused response accuracies to drop to an average of 30.0% for M2^−/−^ and 30.8% for wild-type mice. Accuracy progressively improved over the next sessions and did not differ between wild-type and M2^−/−^ mice (Fig. 1d, RM ANOVA, main effect of session F(11,132) = 8.00, *p* < 0.001; no main effect of genotype, F(1,12) = 3.37, *p* = 0.091), no genotype x session interaction (F(11,132) = 0.45, *p* = 0.93). Although both groups eventually performed above chance (*t* test against 50%: wt, t(7) = 2.4, *p* = 0.043; M2^−/−^, t(5) = 2.8, *p* = 0.047), they never reached performance levels similar to before reversal, which may be related to the feature overlap between the two stimuli.

### Experiment 2: PAL

After visual discrimination, the same cohort of M2^−/−^ (*n* = 8) and wild-type mice (*n* = 9; one animal had to be excluded due to an eye infection) was tested on rodent PAL, which requires the mice to learn to associate a given object with a particular location, an ability impaired in AD patients, after anticholinergic treatment in rodents (Greig et al. 2005; Bartko et al. 2011b), after forebrain depletion of the vesicular acetylcholine transporter (Al-Onaizi et al. 2017) and after application of the M1-receptor-preferring antagonist dicyclomine (Bartko et al. 2011b).

Although choice accuracies gradually increased in both genotypes, M2^−/−^ mice learned significantly more slowly and never reached the accuracy levels of wild-type mice (Fig. 2). A RM ANOVA with genotype as between-subjects factor and block as within-subject factor returned a main effect of genotype (F(1,15) = 4, *p* = 0.046) but no genotype x block interaction (F(11,165) = 1.7, *p* = 0.068), confirming that object-place location learning was significantly impaired in M2^−/−^ mice. A comparison of the mean response latencies between genotypes showed no significant differences, suggesting that the findings were not confounded by procedural or motivational deficits (wt, 3973 ± 410 ms; M2^−/−^, 3525 ± 286 ms; independent-samples *t* test, t(16) = 0.01, *p* = 0.62).

### Experiment 3: 5CSRTT

**Fig. 3:**
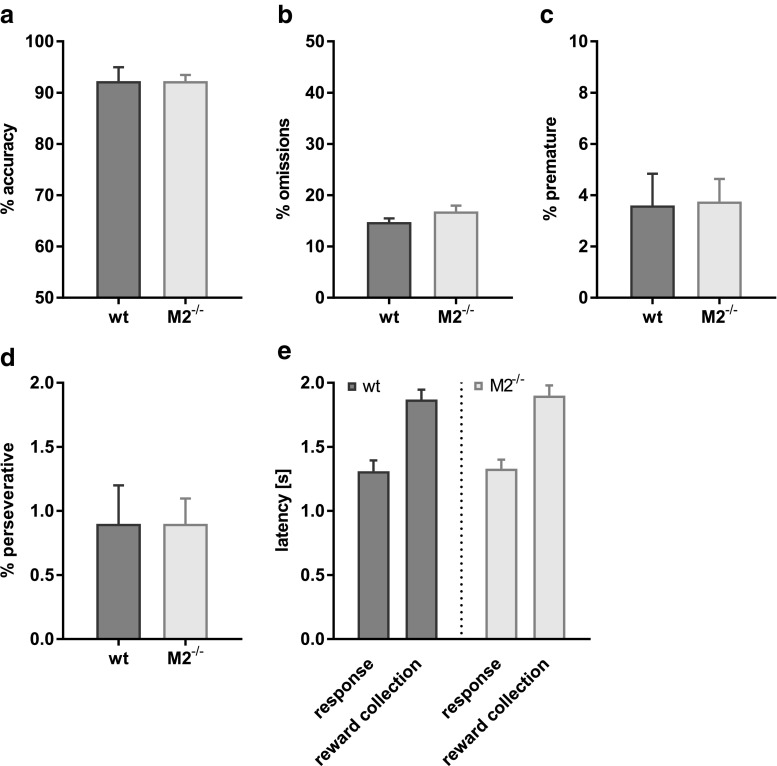
Baseline 5CSRTT performance and control measures of M2^−/−^ mice were unchanged. Baseline **a** choice accuracies (excluding omissions), **b** omissions, **c** premature responses, **d** perseverative responses and **e** response and reward collection latencies at 2 s stimulus duration were similar in wild-type (*n* = 6) and M2^−/−^ mice (*n* = 8). Data are presented as mean ± sem

To assess attention and aspects of executive control, we tested a new cohort of M2^−/−^ (*n* = 8) and wild-type mice (*n* = 6) on the 5CSRTT (Robbins 2002; Dalley and Robbins 2017). The two genotypes required a similar number of sessions to reach the criterion of stable baseline performance (> 80% correct, < 20% omissions at 2 s stimulus duration for 3 of 4 consecutive days; mean sessions to criterion wild-type mice, 18.4 ± 0.8; M2^−/−^, 19.3 ± 0.7; one-way ANOVA, *F* < 1, *p* > 0.1). An analysis of response accuracies, omission errors, response latencies, reward latencies, premature errors and perseverative responses of the last 2 days of baseline performance returned no significant differences between genotypes (Fig. 3a–e, one-way ANOVAs with genotype as between-subjects factor for each measure, all *F* < *p* > 0.1).

**Fig. 4:**
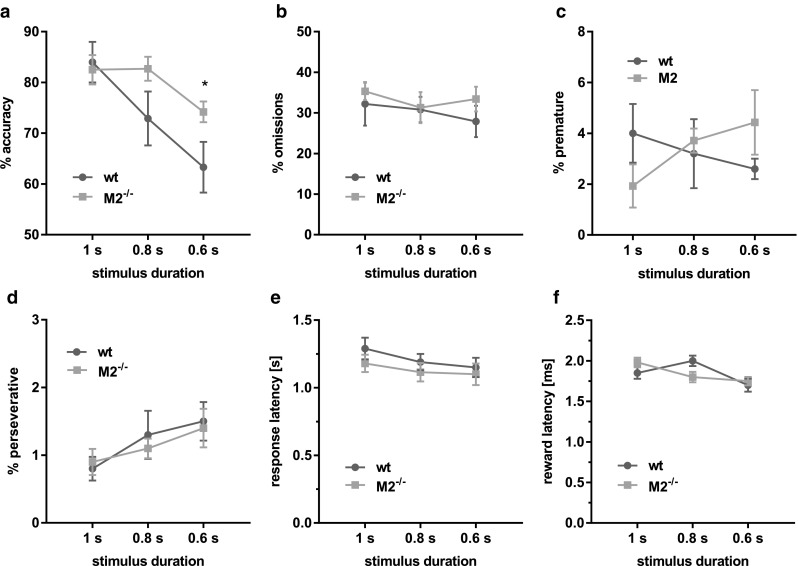
M2^−/−^ mice showed a selective enhancement of accuracy on 5CSRTT probe trials with variable stimulus duration. **a** Choice accuracies (excluding omissions) of M2^−/−^ mice (*n* = 8) were higher than those of wild-type mice (*n* = 6) when stimulus durations were shorter, i.e., attentional demand was increased. Performance was similar to wild-type mice on all other measures of the task (**b**–**e**). Data are presented as mean ± sem. * Simple main effect of genotype at 0.6 s, *p* < 0.05

On the following probe trials, attentional demand was increased by reducing the stimulus duration, i.e., the time the stimulus remained on the screen. Under these circumstances, M2^−/−^ mice made significantly fewer commission errors than wild-type mice, i.e., responded more accurately, especially at shorter stimulus durations (Fig. 4). An RM ANOVA of response accuracies with genotype as between-subjects factor and stimulus duration as within-subject factor returned no main effect of genotype (F(2,24) = 2.8, *p* = 0.12), but a main effect of stimulus duration (F(2,24) = 19.5, *p* < 0.0001) and an interaction of genotype and stimulus duration (F(2,24) = 4.32, *p* < 0.05). A post hoc comparison of simple main effects showed a simple main effect of genotype at 0.6 s (F(1,12) = 7.5, *p* < 0.05), but not at 0.8 s or 1 s stimulus duration (both *F* < 1.2, *p* > 0.05). There were no further significant differences on any other measure of the task (Fig. 4b–f, all *F* < 1, *p* > 0.1).

In order to establish whether the order of stimulus durations on the probe trials affected response accuracies, we analysed mean choice accuracies at 0.6 stimulus duration separately for when the session was run after a baseline session (2 s), or after 1 s, 0.8 s and 0.6 s probe trial sessions, respectively. However, we did not find any significant differences between the conditions, suggesting that there was no major order effect (ANOVA with genotype and session order as between-subjects factors: main effect of genotype F(1,28) = 7.2, *p* = 0.015; no main effect of order or interaction, F(3,28) = 1.2, *p* = 0.34, and F(3,28) = 0.5, *p* = 0.63, respectively).

### Experiment 4: object recognition

Finally, we tested a new cohort of M2^−/−^ and wild-type mice (*n* = 6 for both genotypes) on an object recognition paradigm, a task that had previously revealed impairments in M1^−/−^ mice (Bartko et al. 2014), and requires the functional integrity of the perirhinal cortex (Winters et al. 2004; Forwood et al. 2005). Exploration of the sample object did not differ between genotypes (0 delay: M2^−/−^, 12.5 s ± 4.1 s; wild type, 12.9 s ± 5 s; 3 h delay: M2^−/−^, 13.2 s ± 5.1 s; wild type, 12.7 s ± 3.9 s). A RM ANOVA with genotype as between-subjects factor and delay as within-subject factor revealed no effect of genotype or delay and no interaction (all *F* < 1).

**Fig. 5:**
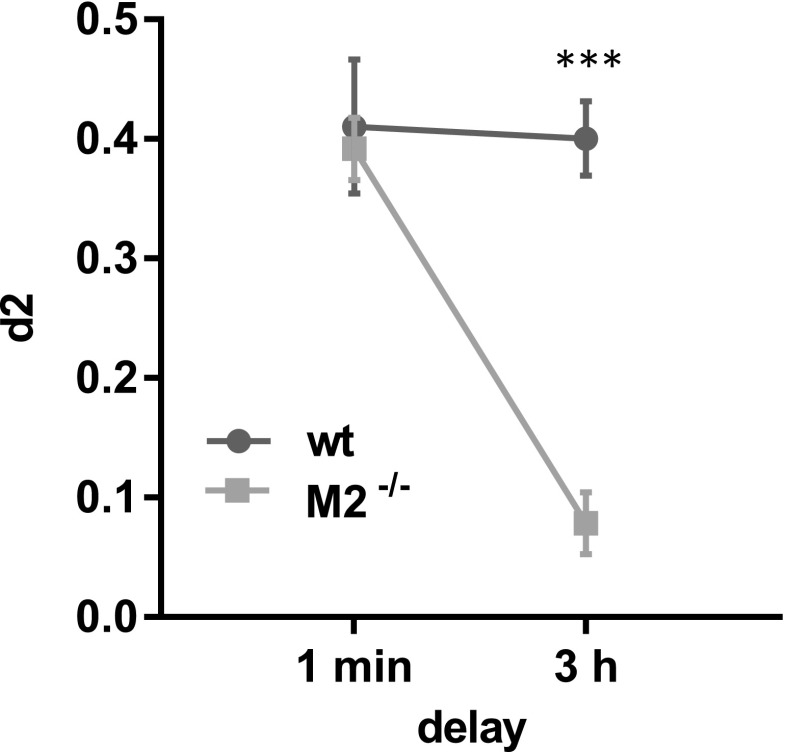
Object recognition is impaired in M2^−/−^ mice. Discrimination scores (d2) of wild type (*n* = 6) and M2^−/−^ mice (*n* = 6) after a delay of 1 min and 3 h. d2 = 0.5 corresponds to an animal exploring the novel object 50% more than the familiar object. Data are presented as mean ± sem. *** Simple main effect of genotype at 3 h delay, *p* < 0.001

During the choice phase, both M2^−/−^ mice and wild-type mice readily discriminated between novel and familiar objects when the delay between sample phase and choice phase was minimally short, i.e., long-term memory was not required, suggesting that M2^−/−^ mice had no perceptual difficulties that could have compromised their overall task performance (Fig. 5). However, M2^−/−^ mice performed dramatically worse than wild-type mice when the delay was long, indicating a profound and specific impairment of memory. A RM ANOVA of d2 scores with genotype as between-subjects factor and delay as within-subject factor returned a main effect of genotype (F(1,10) = 34.1; *p* < 0.001), a main effect of delay (F(1,10) = 12.15; *p* < 0.01) and a genotype by delay interaction (F(1,10) = 10.7; *p* < 0.01), revealing that M2^−/−^ mice performed significantly worse than wild-type animals, when long-term memory was required (post hoc simple main effect of genotype at 3 h, F(1,10) = 72.8; *p* < 0.001; simple main effect of genotype at 0 h, *F* < 1).

## Discussion

Although the behavioural and clinical significance of muscarinic acetylcholine receptors are well-recognised, the effects of specific muscarinic receptor-subtype activation on cognition are still not well understood. The present study aimed to clarify the role of M2 muscarinic receptors in cognition by testing M2^−/−^ mice on multiple translational touchscreen tasks and an object recognition paradigm.

### M2-receptors are required for distinct aspects of memory

The precise role of cholinergic activity for memory is highly debated, and pharmacological studies with anticholinergic agents have yielded conflicting results that may reflect specific roles for individual cholinergic subtypes in distinct aspects of memory (Hasselmo 2006; Ballinger et al. 2016). Yet, the specific role of M2 muscarinic receptors for memory has been difficult to investigate, because truly selective M2 antagonists have been unavailable. Earlier evidence suggested that M2 muscarinic receptor antagonism may enhance memory due to their autoreceptor function, sparking interest as a potential treatment for cognitive disorders related to cholinergic dysfunction, such as AD (Rowe et al. 2003). However, we have shown here that M2 muscarinic receptor activity is important for at least two types of learning and memory tasks: spontaneous object recognition, a test of recognition memory requiring perirhinal cortex (Winters et al. 2004; Forwood et al. 2005), and PAL, a task requiring the formation of object-place associations involving prefrontal, striatal and hippocampal processes (Talpos et al. 2009; Delotterie et al. 2015; Kim et al. 2015; McAllister et al. 2015). In contrast, visual discrimination learning, a test of non-spatial stimulus-reward learning requiring cortico-striatal circuits, and reversal learning, a test of response-inhibition and re-learning (Jones and Mishkin 1972; Bussey et al. 1997), did not require M2 receptors. Thus, M2 receptor activation has heterogeneous effects even within the memory domain, suggesting a dissociation of function depending on the brain region and neuronal populations involved.

#### M2 receptors are not required for stimulus-reward learning

Initially, we analysed M2^−/−^ mouse performance on a visual discrimination task. Visual discrimination learning is a simple, non-spatial associative learning task that requires at least two processes: learning to perceptually discriminate the stimuli and learning which of the two stimuli is associated with reward. Thus, VD-performance is informative about perceptual abilities and stimulus-reward learning, a type of learning depended on orbitofrontal cortex and the dorsal striatum, but largely independent of the medial prefrontal cortex and medial temporal lobe structures (Jones and Mishkin 1972; Bussey et al. 1997; Chudasama and Robbins 2003). We found that M2^−/−^ mice took longer to acquire the initial discrimination with computer-graphic shape stimuli, which may suggest that M2-depletion affected stimulus discrimination or stimulus-reward learning. However, M2^−/−^ mice learned the subsequent, perceptually more demanding task with morphed photographic stimuli as fast as wild-type mice, even when reward contingencies were reversed. Thus, the latter findings provide a clear demonstration of the general ability of M2^−/−^ mice to accurately perceive and discriminate two-dimensional visual stimuli on a computer screen and to associate these with a reward. The initial visual discrimination learning deficit of M2^−/−^ mice may therefore reflect a transient, nonspecific, motivational and/or procedural deficit, rather than a true perceptual impairment or a stimulus-reward learning deficit.

Thus, although cholinergic signalling from the basal forebrain is thought to be involved in the fine-tuning of sensory cortical representations required for stimulus discrimination (Hasselmo and McGaughy 2004; Hasselmo and Giocomo 2006; Herrero et al. 2008; Goard and Dan 2009; Chen et al. 2015), M2 muscarinic receptors may not be an essential subtype for these processes. Furthermore, the results suggest that M2 receptors do not significantly contribute to stimulus-reward association learning and the underlying neural processes in the striatal-thalamocortical pathway. Interestingly, patients with mild cognitive impairment and early-stage AD, conditions accompanied by a loss of cortical M2 receptors (Mash et al. 1985), were also unimpaired on a similar touchscreen-operated test (Lee et al. 2007).

#### M2 receptors are not required for reversal learning

We also found that M2 depletion had no effect on reversal learning in either of the two discrimination tasks. The reversal phase is considered to represent two distinct processes: animals have to learn to inhibit pre-potent responses to previously correct stimuli, which is the dominant cognitive process during the initial reversal phase and mediated by medial PFC structures (Chudasama et al. 2001; Chudasama and Robbins 2003). The later stage of reversal learning is dominated by the re-learning of the new reward-contingencies, and as such dependent on thalamocortico-striatal interactions, like the original visual discrimination task. Our data show that M2^−/−^ mice learned to inhibit the previously acquired stimulus-reward association at the same rate as wild-type mice. However, because M2^−/−^ mice took longer to acquire the initial discrimination (although, note that the cumulative percentage of animals that had reached criterion was similar in both genotypes), it is possible that a weaker stimulus-reward association may have facilitated the first reversal, masking a potential reversal deficit. Yet, we think this is unlikely, since baseline accuracies before reversal were similar in both genotypes, suggesting that M2^−/−^ mice had eventually formed stimulus-reward associations similar to wild-type mice. Moreover, M2^−/−^ mice reversed as quickly as wild-type mice after the second visual discrimination, which they had required at the same rate as wild-type mice. Thus, we found no compelling evidence that M2 receptors contribute to flexible adaptation to new reward contingencies.

M2^−/−^ mice also performed similar to wild-type mice on the later phase of both reversal tasks, confirming that M2-depletion did not affect (re)-learning of new stimulus-reward associations. However, performance levels after the second reversal never reached pre-reversal accuracies, which may relate to the considerable feature overlap of the two morphed photographic stimuli, resulting in more pro-active interference from the previous association. Nevertheless, both genotypes eventually performed above chance, indicating that a significant reversal learning had occurred.

Although our data suggest that M2 receptors are not required for reversal learning, M2 muscarinic receptors may be required for cognitive flexibility when the task has a spatial component: M2^−/−^ mice were impaired on a spatial reversal task on the Barnes maze (Seeger et al. 2004). The different outcome on these reversal learning tasks is interesting and may reflect functionally distinct roles of M2 receptors in hippocampus and prefrontal cortex that may be related to distinct cellular/subcellular locations of M2 receptors in the respective areas. To some extent, the differentiation of impaired spatial versus intact non-spatial reversal learning in M2^−/−^ mice is relevant to the ongoing debate regarding the role of cholinergic afferents from the basal forebrain to the prefrontal cortex and hippocampus in spatial memory: several behavioural studies employing IgG-192 saporin cholinergic lesions indicate a potential role of cholinergic innervation in forming representations of spatial location relevant to memory-guided behaviour, rather than spatial memory per se (Baxter et al. 1995; Parent and Baxter 2004; Cai et al. 2012; Ballinger et al. 2016).

#### M2-depletion impairs stimulus-place association learning

M2^−/−^ mice were profoundly impaired on PAL, a visuospatial associate memory task that has similar perceptual demands to visual discrimination, but ideally cannot be solved with a simple stimulus-reward learning strategy. Instead, it requires the formation of three individual stimulus-location associations and is sensitive to hippocampal and prefrontal cortical manipulation both in rodent and humans (Talpos et al. 2009; Kim et al. 2015; McAllister et al. 2015).

Although PAL may theoretically be solved with a conditional learning rule associating each of the 6 possible trial types with a response (e.g., if trial type 1 touch left, if trial type 2 touch middle,…), we previously found that this was not the case: neither did an additional stimulus in the neutral location have a greater impact on PAL performance (sPAL, Talpos et al. 2009) nor was the mean performance biased towards individual trial types (Talpos et al. 2009; McAllister et al. 2015). Therefore, PAL seems to be predominantly solved with a stimulus-in-location strategy in both humans and rodents (Nithianantharajah et al. 2015).

The PAL deficit of M2^−/−^ mice became apparent early during the acquisition process and persisted until the end of testing, suggesting that M2 muscarinic receptors are also important for object-in-place learning. PAL and other types of object-in-place learning are sensitive to manipulation of the medial prefrontal cortex (Kesner and Ragozzino 2003; Browning et al. 2005; Barker et al. 2007; Barker and Warburton 2009; McAllister et al. 2015), but also involve striatal and hippocampal processes (Delotterie et al. 2015; Kim et al. 2015). However, hippocampal manipulations most strongly affect PAL performance, with only mild effects on acquisition (Hernandez et al. 2015; Kim et al. 2015), and the rodent striatum largely lacks M2 muscarinic receptors (Zhang et al. 2002). Thus, the PAL deficit of M2^−/−^ mice may reflect medial prefrontal cortex (mPFC) dysfunction, suggesting that M2^−/−^ receptors in mPFC are involved in the neural processes supporting object-in place learning. Interestingly, a muscarinic form of LTD akin to perirhinal LTD has also been described in mPFC (Caruana et al. 2011). Moreover, M2-receptors are essential for a form of acetylcholine-induced LTD at hippocampal-mPFC synapses (Wang and Yuan 2009)**.**

#### M2 receptors are required for object recognition memory

Furthermore, we found that dysfunction of M2 receptors profoundly impaired object recognition memory, a type of one-trial learning with neural correlates in perirhinal cortex (Winters et al. 2008). The impairment was specific to the task version with a 3-h delay, whereas M2^−/−^ mice had no difficulties on the object recognition task when the delay was very short. Thus, M2 depletion leaves novelty detection and object perception per se intact, but specifically impairs the memory component of object recognition, a pattern we also found after perirhinal infusion of the M2 receptor-preferring muscarinic antagonist AF-DX 116 in wild-type mice (Bartko et al. 2014).

Neural correlates of object recognition that engage M2 muscarinic receptors remain to be demonstrated, but intra-perirhinal infusion of scopolamine in rats impaired object recognition and also impaired long term depression (LTD) in cortical slices (Warburton et al. 2003; Winters et al. 2006), suggesting that a muscarinic receptor-dependent form of perirhinal LTD contributes to object recognition. Although earlier studies suggest that this type of plasticity requires M1 muscarinic receptor activation (Massey et al. 2001; Warburton et al. 2003), the role of M2 muscarinic receptors in perirhinal synaptic plasticity has not been directly investigated. However, M2 receptors have been found to contribute to muscarinic forms of synaptic plasticity in the hippocampus (Seeger et al. 2004; Zheng et al. 2012), at hippocampal-prefrontal synapses (Wang and Yuan 2009) and in the medial prefrontal cortex (Caruana et al. 2011). Thus, an obvious explanation for the detrimental effect of M2 depletion on OR may be that M2 receptors contribute to perirhinal cortical LTD or other forms of synaptic plasticity.

Thus, although M2 muscarinic receptor inactivation may benefit some cognitive abilities through enhancing cholinergic tone, our and other recent data suggests that impaired M2-signalling may compromise memory processes (Seeger et al. 2004; Bainbridge et al. 2008; Bartko et al. 2014).

### M2 receptor dysfunction enhances sustained attention

In contrast to the partial memory deficits described above, M2 receptor dysfunction lead to an enhancement of sustained attention on the 5CSRTT. Accuracy on the 5CSRTT is sensitive to cholinergic manipulation (Robbins 2002; Chudasama et al. 2004; Dalley et al. 2004). Basal forebrain lesions (Muir et al. 1994), cholinergic denervation of the prefrontal cortex (McGaughy et al. 2002), depletion of the forebrain vesicular acetylcholine transporter (Kolisnyk et al. 2013; Al-Onaizi et al. 2017) and administration of scopolamine, a muscarinic antagonist (Jones and Higgins 1995; Humby et al. 1999; Mirza and Stolerman 2000) can all lead to selective accuracy deficits on the 5CSRTT. However, at least for scopolamine, the data are inconsistent (Mirza and Stolerman 2000; Robbins 2002) and it has not been established through which cholinergic receptor subtypes these effects are mediated. The findings of the present study indicate that deficits following such manipulations may not be due to impaired M2 muscarinic receptor function. Instead, we show here that in the absence of M2 muscarinic receptor activity, choice accuracy is in fact enhanced, without any changes to other measures of 5CSRTT performance, such as premature and perseverative responses, response and reward latencies, or omissions. In particular the unchanged omission errors imply that target detection and related visual processes were unaffected, suggesting that M2 deficiency caused a true enhancement of sustained attention.

An obvious explanation for the facilitating effect of M2 depletion on sustained attention may be an increase of cholinergic responses within the prefrontal cortex, since M2 muscarinic receptors are predominantly found as pre-synaptic autoreceptors attenuating ACh efflux from basal forebrain efferents (Zhang et al. 2002). Although the effects of M2-depletion on ACh in prefrontal cortex have not been studied directly and may be distinct from the hippocampus, a pronounced increase of the phasic ACh response in response to a novel environment was observed in the hippocampus of M2^−/−^ mice, while basal ACh levels remained unchanged (Tzavara et al. 2003; Thomsen et al. 2018). A similar transient increase of ACh efflux is observed in the prefrontal cortex during 5CSRTT performance and related to attentional effort, in particular in the face of performance challenges (Dalley et al. 2001). Thus, the enhancement of sustained attention in M2^−/−^ mice may relate to an enhanced transient ACh response. In contrast, a general increase of postsynaptic ACh by cholinesterase inhibitors seems to increase response accuracies only when those are compromised due to other manipulations, such as basal forebrain lesions or AD pathology (Muir et al. 1995; Romberg et al. 2011). Similarly, mice lacking the vesicular acetylcholine transporter in the forebrain show no changes in response accuracy on the 5CSRTT, although they present decreased vigilance and increased omission (Kolisnyk et al. 2013).

Furthermore, in addition to their role as autoreceptors on ascending cholinergic neurons, heterosynaptic M2 receptors are also found on local glutamatergic and GABAergic neurons in medial prefrontal cortex (Volpicelli and Levey 2004). Thus, M2-depletion may also affect the excitatory/inhibitory balance of cortical structures, which may contribute to changes in attentional state.

### Why does M2-depletion have opposing effects on attention and memory?

Although highly speculative, our data may also imply that M2 receptors serve distinct functional roles in cognition, depending on the neuronal populations by which they are expressed. Presynaptic M2 receptors on ascending cholinergic neurons modulate the availability of ACh at their neuronal target populations (Zhang et al. 2002).

Thus, M2 depletion on ascending cholinergic neurons may lead to a phasic or tonic increase of ACh in cortical regions, which may enhance some aspects of cognition, such as attention (see previous section), but may be less favourable for certain types of memory (Bunce et al. 2004; Hasselmo and McGaughy 2004; Gais and Born 2004; Picciotto et al. 2012; Kolisnyk et al. 2013; Al-Onaizi et al. 2017). Moreover, M2 receptors are also expressed on GABAergic interneurons in cortical/hippocampal target structures (Volpicelli and Levey 2004; Seeger et al. 2004; Ballinger et al. 2016), where they modulate the degree and timing of inhibition, and may contribute to synaptic plasticity (Hájos et al. 1998; Seeger et al. 2004; Bainbridge et al. 2008; Wang and Yuan 2009; Caruana et al. 2011). Thus, M2 receptors may function both as autoregulators of cholinergic projection neurons (M2 depletion increases cholinergic tone and enhances attention) and as regulators of local GABAergic inhibition, changing how basal forebrain target areas respond to ACh and other input (M2-depletion increases GABAergic inhibition, impairing synaptic plasticity and memory)*.* Further experiments will have to clarify how M2 muscarinic receptors expressed in different neuronal subgroups, i.e., within GABAergic, glutamatergic and cholinergic neuronal populations, act in concert to contribute to cognitive control.

## Conclusions

Overcoming previous limitations of non-selective M2 receptor antagonists and cognitive tests focusing on only one cognitive domain, we have shown that dysfunction of M2 receptors leads to improvements in sustained attention—in the face of impairments in learning and memory (also see Bainbridge et al., 2008 for the latter). Thus, M2 receptor activation has heterogeneous effects across different cognitive domains. Moreover, we demonstrate that M2 receptor function even varies within the memory domain: complementing previous work reporting spatial reference memory and spatial reversal learning deficits (Bainbridge et al. 2008), we show that M2 receptors are required for associative stimulus-location learning and object recognition, but do not contribute to visual discrimination and reversal learning. Thus, we conclude that the role of M2 receptors in cognition may depend on the brain region involved and/or a functional dissociation within brain regions, possibly depending on the neuronal cell-type M2 receptors are expressed in. Specifically, M2 receptor activation within the mPFC may be beneficial for stimulus-location association learning and object recognition, perhaps by facilitating synaptic plasticity through local M2 receptors on glutamatergic and GABAergic neurons. In contrast, M2 receptor activation is detrimental to sustained attention, perhaps by limiting cholinergic activation in response to external stimuli. Intriguingly, the variable effects of M2-depletion across and within different cognitive domains are consistent with the influential view that a high cholinergic tone sets the circuit dynamics for attention and memory encoding, whereas low ACh levels facilitate memory consolidation and retrieval (Hasselmo and McGaughy 2004).

Furthermore, the results suggest that despite current interest in therapeutics involving M2 receptor-active compounds, such compounds should be assessed across a broad range of cognitive domains, as they may enhance some cognitive functions, but impair others.
